# Genetically Distinct African Swine Fever Virus Genotype II Variant in Wild Boar, Spain, 2025–2026

**DOI:** 10.3201/eid3209.260360

**Published:** 2026-09

**Authors:** Carmina Gallardo, Marina Marcet-Houben, Montserrat Agüero, Raquel Nieto, Nadia Casado, Alejandro Soler, María José Ruano, Rubén Villalba, Jovita Fernández-Pinero, Alicia Simón, Covadonga Pérez, Concepción Gómez, Cecilia García, Mireia Labrador, Jordi Argilaguet, Liani Coronado, Natàlia Majó, Pilar Aguilera-Sepulveda, Irene Aldea, Toni Gabaldón, María Belén Gómez, Marisa Arias

**Affiliations:** Centro de Investigación en Sanidad Animal, Valdeolmos, Spain (C. Gallardo, R. Nieto, N. Casado, A. Soler, J. Fernandez-Pinero, A. Simón, C. Pérez, C. Gómez, P. Aguilera-Sepulveda, I. Aldea, M. Arias); Barcelona Supercomputing Center, Barcelona, Spain (M. Marcet-Houben, C. García, M. Labrador, T. Gabaldón); Institute for Research in Biomedicine, Barcelona (M. Marcet-Houben, C. García, M. Labrador, T. Gabaldón); CIBER de Enfermedades Infecciosas, Instituto de Salud Carlos III, Madrid, Spain (M. Marcet-Houben, T. Gabaldón); Laboratorio Central de Veterinaria, Algete, Spain (M. Agüero, M.J. Ruano, R. Villalba, M.B. Gómez); Unitat Mixta d’Investigació Institut de Recerca i Tecnologia Agroalimentàries–Universitat Autònoma de Barcelona en Sanitat Animal, Barcelona (J. Argilaguet, L. Coronado, N. Majó); Centre de Recerca en Sanitat Animal, Barcelona (J. Argilaguet, L. Coronado); World Organisation for Animal Health Collaborating Centre for the Research and Control of Emerging and Re-Emerging Swine Diseases in Europe, Barcelona (J. Argilaguet, L. Coronado, N. Majó); Universitat Autónoma de Barcelona, Barcelona (N. Majó); Catalan Institution for Research and Advanced Studies, Barcelona (T. Gabaldón)

**Keywords:** African swine fever virus, viruses, genotype II, wild boar, whole-genome sequencing, multigene typing, Spain, left variable region

## Abstract

African swine fever virus genotype II was detected in wild boar in Spain in 2025 after >30 years of absence. Whole-genome sequencing and molecular follow-up of 8 representative cases identified a stable 9.8-kb deletion affecting the multigene family in the left variable region, defining an ASFV genotype II variant.

African swine fever (ASF) is a highly contagious disease of domestic pigs and wild suids with major economic consequences. After the emergence of ASF virus (ASFV) genotype II in Georgia in 2007 (*1*), the virus spread across Europe and Asia and reached the Caribbean, demonstrating its capacity for transcontinental spread. In Europe, ASFV circulation is maintained in wild boar populations (*2*). On November 26, 2025, ASFV was confirmed in Spain (*3*). By May 29, 2026, a total of 326 ASFV-positive wild boar had been detected (Figure 1, panel A). Spain rapidly implemented control measures to limit wild boar movement, including carcass search and removal, reinforced passive surveillance, movement restrictions, and installation of fencing. This study describes the genomic characterization and molecular follow-up of 8 representative ASFV-positive wild boar cases detected during November 2025–February 2026, including the index cases (Figure 1, panel B).

**Figure 1:**
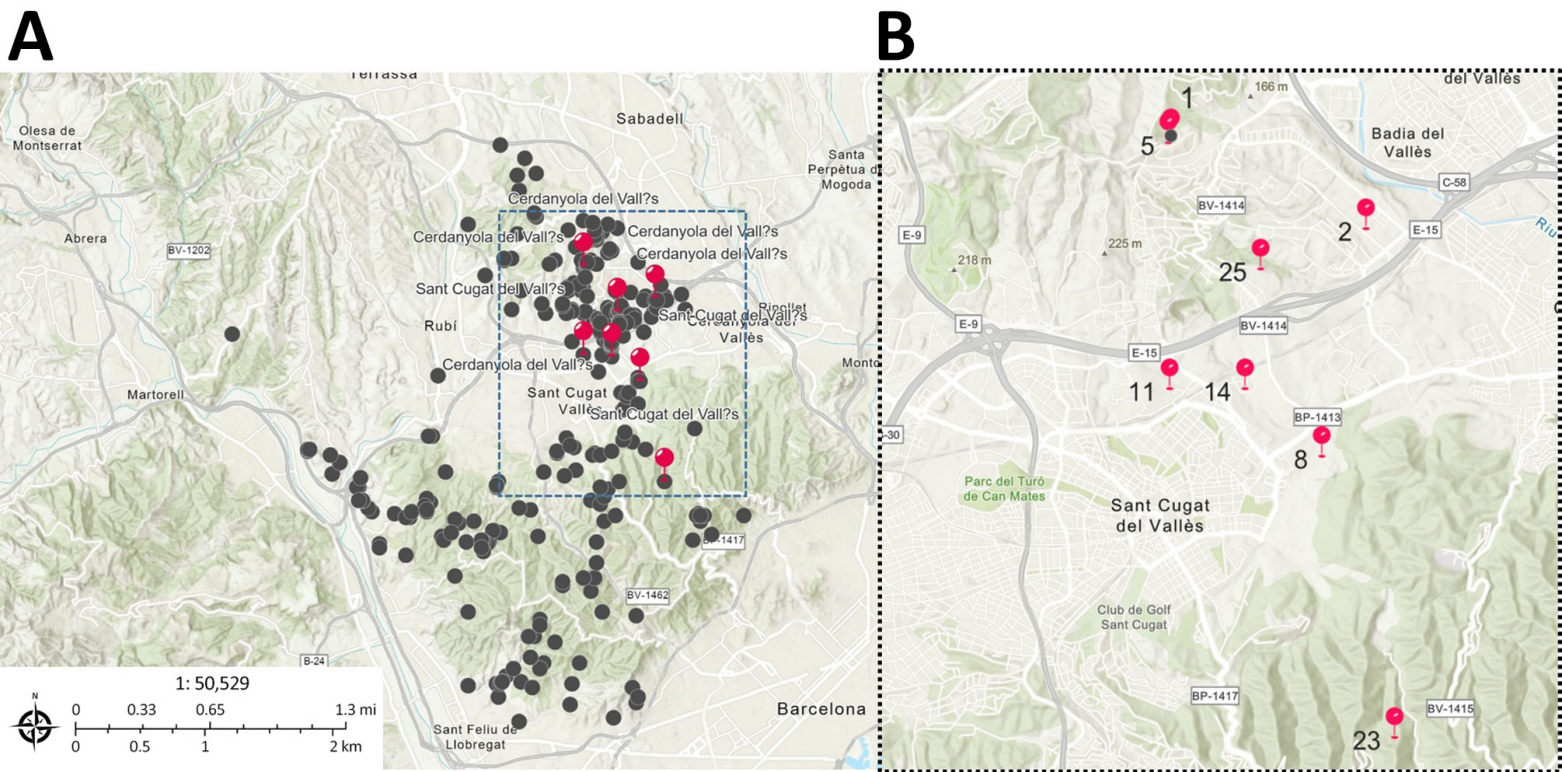
Spatial distribution of African swine fever virus (ASFV)–positive wild boar cases detected in northeastern Spain, 2025–2026. A) Geographic distribution of ASFV-positive wild boar cases detected during November 2025–May 2026 in the affected area in Barcelona. Red dots indicate cases selected for whole-genome sequencing. B) Enlarged view of the main affected area showing the spatial distribution of sequenced ASFV-positive wild boar cases. Numbers correspond to genome identifiers: 1, SP25WBCASE1 (index case); 2, SP25WBCASE2 (index case); 5, SP25WBCASE5 (case 0); 8, SP26WBCASE8; 11, SP26WBCASE11; 14, SP26WBCASE14; 23, SP26WBCASE23; 25, SP26WBCASE25.

## The Study

Two freshly dead wild boar (*Sus scrofa*) were found on November 26, 2025, during passive wildlife surveillance near Barcelona, northeastern Spain (*3*). The animals were located ≈1 km apart in a periurban area with high human connectivity. We analyzed whole blood, serum, spleen, tonsil, and lymph node samples at Unitat Mixta d’Investigació Institut de Recerca i Tecnologia Agroalimentàries, Centre de Recerca en Sanitat Animal (IRTA-CReSA; Bellaterra, Spain), the Spanish National Reference Laboratory for ASF (Algete, Spain), and the European Union Reference Laboratory for ASF (EURL-ASF; Valdeolmos, Spain).

We confirmed that both animals tested ASFV-positive by World Organisation for Animal Health (WOAH) real-time PCR procedure 2; virus isolation in primary porcine alveolar macrophages showed the characteristic hemadsorption pattern. We detected ASFV-specific antibodies in both animals by WOAH indirect immunoperoxidase test; serum titers were 1:2,560.

**Figure 2:**
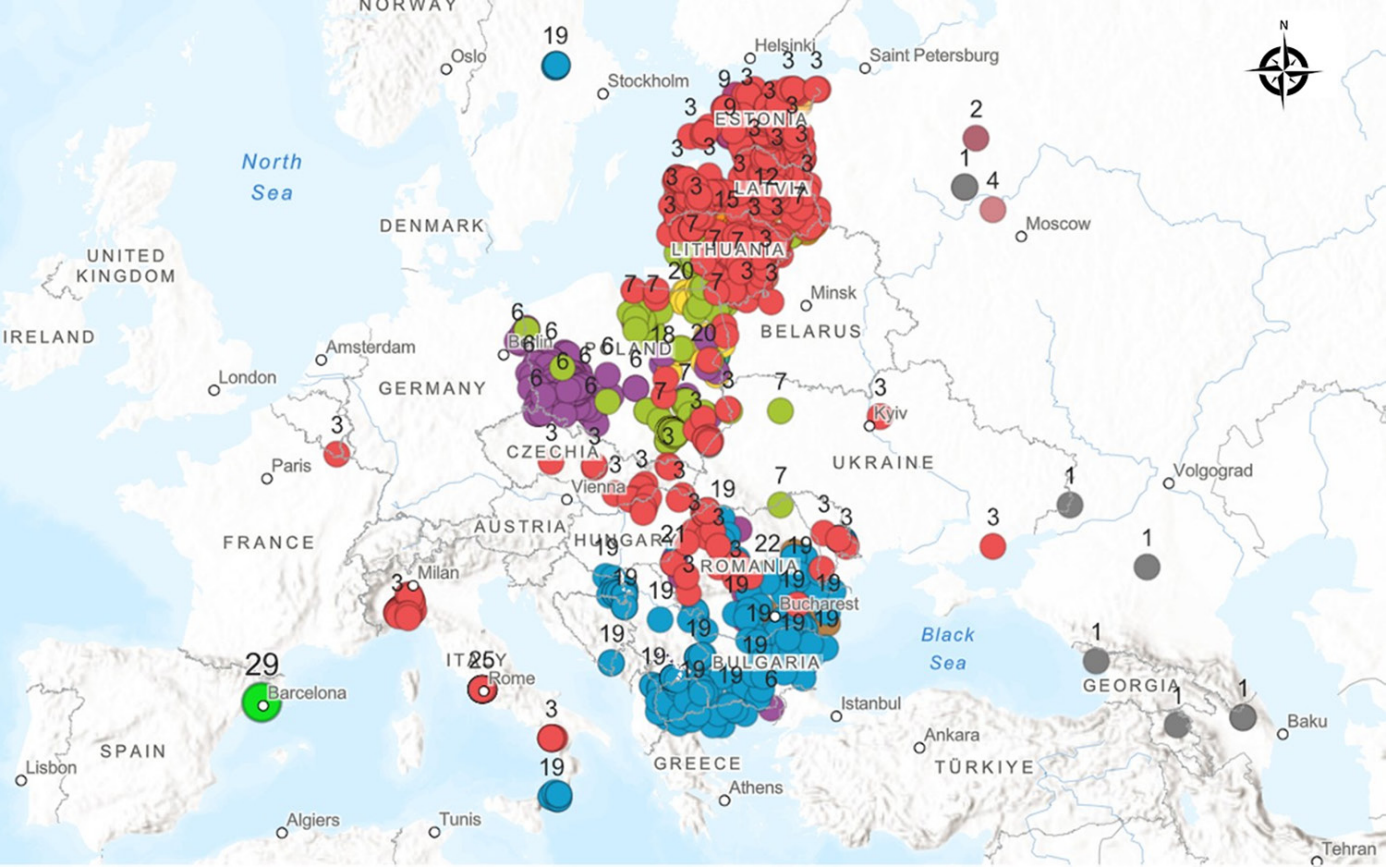
Geographic distribution of African swine fever (ASF) virus genotype II genetic groups in Europe, 2007–2025. We combined analysis of 6 genomic regions to define genetic groups. We assigned groups through comparison with 1,192 homologous genotype II sequences available in the European Union Reference Laboratory for ASF sequence database (Appendix 1 Table 1). Numbers within colored circles denote genetic groups. Genetic group 29, identified in Spain in 2025, is highlighted. The analyzed regions were the central variable region within B602L; intergenic region (IGR) between I73R and I329L genes; O174L; K145R; the IGR between MGF (multigene family) 505-9R and MGF505-10R genes; and the IGR between I329L and I215L genes.

Genotyping based on partial *B646L* (p72) sequencing assigned the virus to genotype II, which has circulated in Europe since 2007 (*4*). To determine whether the Spain ASFV corresponded to any of the 28 genotype II genetic groups the EURL-ASF identified in Europe (Figure 2), we analyzed viruses from both index wild boar using a harmonized 6-locus framework (*5*). Comparison with 1,192 homologous genotype II sequences available in the EURL-ASF sequence database (Appendix 1 Table 1) showed that 5 of 6 analyzed loci matched the Georgia 2007 genotype II variant 1 profile, whereas a previously undescribed G→A substitution within the MGF505-*9R/10R* intergenic region supported classification of the Spain isolate as a new genetic group, 29 (Figure 2).

We performed whole-genome sequencing to obtain higher-resolution genomic characterization of the Spain ASFV. We processed ASFV-positive blood samples using target-enriched libraries prepared with KAPA HyperCap workflow (Roche Sequencing Solutions, https://diagnostics.roche.com) and sequenced on an Illumina iSeq 100 platform (https://www.illumina.com), generating 2 × 150 bp paired-end reads. We mapped reads to the ASFV Georgia 2007/1 reference genome (GenBank accession no. FR682468.2). Both index wild boar cases yielded nearly identical genomes of 180,757 nt; mean coverage exceeded 200 times. We designated the consensus genome sequence SP25WB2611 (GenBank accession no. PZ023911).

Sequence analysis revealed a ≈9.8-kb deletion within the left variable region (LVR), spanning positions 10,264–20,087 of the reference genome. The deletion removed 21 coding sequences, predominantly members of multigene family (MGF) 110, together with MGF360-4L, MGF360-6L, and MGF100-1R (Appendix 2 Figure). Outside the deleted region, the genome retained >99.9% homology with Georgia 2007/1 with 30 differences: 18 single-nucleotide polymorphisms (SNPs) and 12 insertions or deletions.

**Figure 3:**
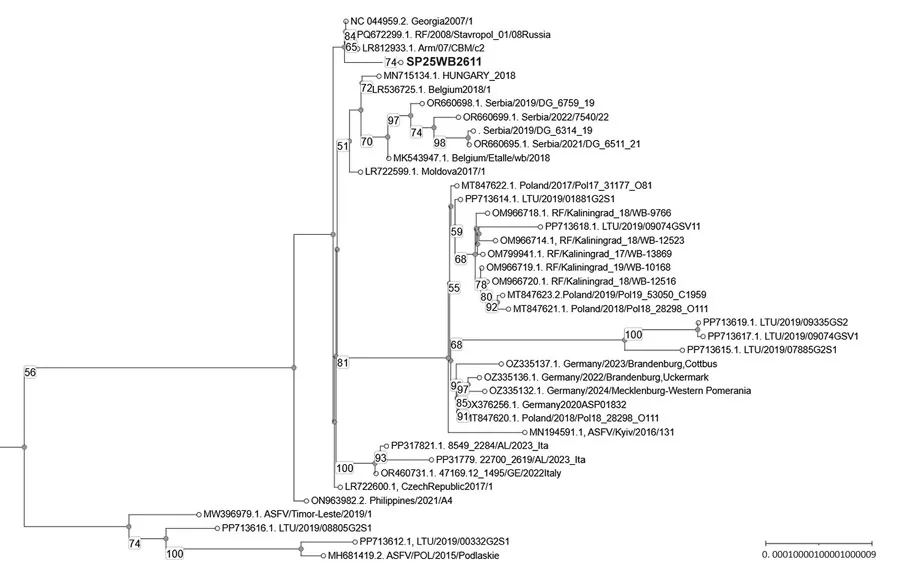
Maximum-likelihood phylogenetic tree based on complete genome sequences of ASFV genotype II from study of the variant in wild boar, Spain, 2025–2026. The analysis included 40 publicly available genomes retrieved from GenBank (accession numbers shown) and the consensus genome sequence SP25WB2611 from our study (bold; wild boar, Spain, 2025 case 1 and 2). We used IQ-TREE (http://www.iqtree.org) to infer the tree with the best-fitting nucleotide substitution model selected by ModelFinder (http://www.iqtree.org/ModelFinder) outside the variable regions. Branch support values are shown at nodes (10,000 ultrafast bootstrap replicates). Scale bar indicates nucleotide substitutions per site. ASFV, African swine fever virus.

To investigate the phylogenetic relationship of the Spanish ASFV, we retrieved 40 genotype II genomes sharing >99.94% nucleotide identity with the Spain isolate from GenBank. Maximum-likelihood analysis placed SP25WB2611 within the major genotype II lineage derived from Georgia 2007/1 (Figure 3). The Spain isolate clustered near historical Caucasus genotype II viruses, including Armenia 2007 and the Russian Federation 2008 Stavropol isolate, on the basis of SNP similarity outside the large LVR deletion.

To support molecular follow-up of the outbreak, we selected 6 additional ASFV-positive wild boar cases detected during November 2025–February 2026 for whole-genome sequencing (Figure 1, panel B; Appendix 1 Table 2). Those included the oldest ASFV-positive case identified during field investigation (SP25WBCASE5), considered the probable index case of the outbreak (case 0), which had likely been dead for ≈1.5–4 months before detection. All genomes clustered within the same clade and showed the characteristic ≈9.8-kb LVR deletion and nearly identical profiles compared with SP25WB2611. The only relevant difference was a synonymous SNP in case 11 relative to the Georgia 2007/1 reference genome (C→T at position 164,421). We also observed minor variations within polynucleotide tracts. We described genomic variants and epidemiologic data (Appendix 1 Tables 2, 3). We submitted the 6 consensus genome sequences to GenBank (accession nos. PZ619929–34). 

## Conclusions

Genomic characterization of ASFV detected in wild boar in northeastern Spain identified a distinct genotype II variant defined by a unique multigene profile and a large LVR deletion. Outside the deleted region, although the virus remained highly similar to the Georgia 2007/1 lineage, we found 30 nt differences, together with the novel MGF signature defining genetic group 29. In whole-genome phylogenetic analysis, the study isolate clustered near early Caucasus genotype II viruses. However, that clustering should be interpreted cautiously because the large LVR deletion contributed little to the phylogenetic topology. Furthermore, genotype II ASFVs show limited genomic divergence because of their slow evolutionary rate (≈1–1.5 × 10^−5^ substitutions/site/year; ≈2 substitutions/genome/year) (*6*). Consequently, relatively few SNP differences might influence phylogenetic grouping among closely related genotype II viruses. The clustering we observed does not necessarily indicate a direct epidemiologic relationship with early Caucasus isolates.

Analysis of 6 additional ASFV-positive wild boar cases confirmed the stability of the large LVR deletion and the high genomic similarity among the Spain isolates; we detected 1 synonymous SNP within the *E423R* coding region in case 11. Of importance, case 0 already carried the same deletion, suggesting that it was present before introduction into Spain. Although the precise origin of the variant remains unresolved, our data support a long-distance, human-mediated introduction; the isolated detection in a periurban setting with high human connectivity, along with the absence of epidemiologic continuity with affected regions, support that interpretation. Contaminated pork products remain a recognized pathway for ASFV introduction (*7*–*9*).

The genomic features of the Spain ASFVs result primarily from structural changes within the LVR, particularly the deletion affecting MGF110 and MGF360 genes involved in host–virus interactions and innate immune responses (*10*,*11*). Comparable LVR deletions have been reported in ASFV field variants from Estonia and Italy with different virulence outcomes (*12*,*13*), during adaptation of genotype II viruses to continuous cell cultures (*14*), and after heat treatment (*15*). In the Spain variant, the biological consequences of such changes remain uncertain. Serologic findings during outbreak follow-up at the NRL identified ASFV-specific antibodies in 44 (32.1%) of 137 PCR-positive wild boar specimens, confirming the initial findings in the index cases. Those data suggest a more prolonged course of infection than typically observed with highly virulent genotype II ASFV strains, in which animals often die within the first week after infection, before developing detectable humoral responses. Whether the prolonged course of infection we observed in this outbreak is associated with the large LVR deletion requires confirmation through in vivo studies. Our study highlights the importance of continued epidemiologic and genomic follow-up of ASFV outbreaks to better understand the evolution, circulation, and emergence of genetically distinct genotype II variants over time.

Appendix 1 Data from study of genetically distinct African swine fever virus genotype II variant in wild boar, Spain, 2025–2026.

Appendix 2 Additional information about genetically distinct African swine fever virus genotype II variant in wild boar, Spain, 2025–2026.
