# Supplementary material for: Genetically Distinct African Swine Fever Virus Genotype II Variant in Wild Boar, Spain, 2025–2026
Source: Emerg Infect Dis. 2026 Sep;32(9):1497–501. doi: 10.3201/eid3209.260360 (PMC13555704; doi:10.3201/eid3209.260360)
Supplement: Supplementary file 1 [file 26-0360-Techapp-s2.pdf]

Article DOI: https://doi.org/10.3201/eid3209.260360

EID cannot ensure accessibility for supplementary materials supplied by authors. Readers who have difficulty accessing supplementary content should contact the authors for assistance.

Genetically Distinct African Swine Fever Virus Genotype II Variant in Wild Boar, Spain, 2025–2026

Appendix 2

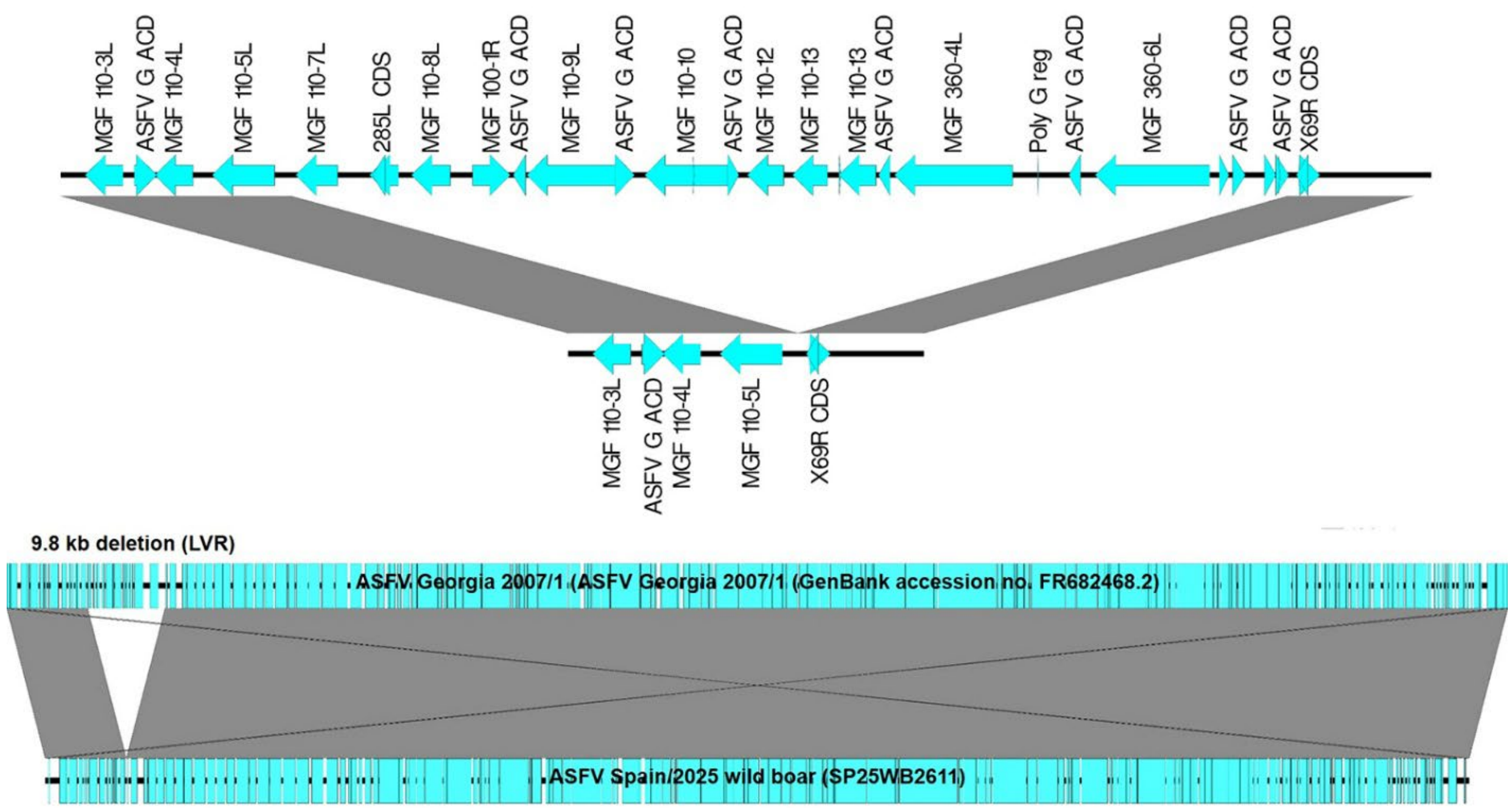

Appendix 2 Figure. Genetic distinctions in African swine fever virus genotype II isolates from wild boar, Spain, 2025–2026. Deletion in LVR of the African swine fever virus isolate Spain/2025 (SP25WB2611) compared with reference strain Georgia 2007/1 (GenBank accession no. FR682468.2). The Spain/2025 genome contains a 9.8-kb deletion (positions 10,264–20,087 relative to the reference genome), resulting in loss of 21 coding sequences, including multiple MGF110 genes and additional MGF genes. The lower section shows whole-genome collinearity analysis demonstrating overall genomic conservation and absence of large-scale rearrangements. ASFV, African swine fever virus; CDS, coding sequence; LVR, left variable region; MGF, multigene family.
